# Analysis of the Clinical Characteristics of Spontaneous Bile Duct Perforation in Children

**DOI:** 10.3389/fped.2022.799524

**Published:** 2022-03-23

**Authors:** Xueqiang Yan, Nannan Zheng, Jinfu Jia, Houfang Kuang, Haiyan Lei, Hongqiang Bian, Xinke Qin, Xuan Sun, Xufei Duan, Jianghua Zhan

**Affiliations:** ^1^Department of General Surgery, Wuhan Children's Hospital, Tongji Medical College, Huazhong University of Science and Technology, Wuhan, China; ^2^Department of CT and MRI, Wuhan Children's Hospital, Tongji Medical College, Huazhong University of Science and Technology, Wuhan, China; ^3^Department of Pediatric Surgery, Fujian Provincial Hospital, Fuzhou, China; ^4^Tianjin Children's Hospital, Tianjin, China

**Keywords:** biliary tract, perforation, common bile duct dilatation, pediatric surgery, spontaneous bile duct perforation, pancreaticobiliary malunion

## Abstract

**Objective:**

This study aimed to explore the etiology, clinical features, diagnosis, and treatment of spontaneous bile duct perforation (SBDP) in children.

**Methods:**

The clinical data of children with SBDP who were admitted to Wuhan Children's Hospital between January 2014 and January 2020 were retrospectively analyzed.

**Results:**

In all, 28 cases of children with SBDP (male, 28.6%; female, 71.4%; male-to-female ratio, 1:2.5; average age, 2.15 years) were analyzed. The most common symptoms were fever (85.7%), nausea and vomiting (78.6%), and abdominal distension (67.9%). Among the 28 patients, 26 (92.9%) had elevated hypersensitive C-reactive protein, 24 (85.7%) had an increased neutrophil percentage, and 22 (78.6%) had raised peripheral blood leukocyte counts. Moreover, 19 patients (67.9%) showed increased serum total bilirubin levels, and 5 (17.9%) showed an elevated conjugated bilirubin level. Abdominal CT examination revealed that the gallbladder wall of patients was thickened with edema, accompanied by gallbladder stenosis and gallbladder mucosa enhancement; furthermore, ascites was found in the abdominal cavity and lesser omental bursa. Twenty-two patients underwent abdominal paracentesis, and 20 (90.9%) of them were exposed to bile-based ascites. Among the 28 patients, four recovered with conservative treatment, whereas the others (85.7%) were surgically treated. Of the twenty-four patients undergoing surgery, the perforation site was found at the union of the hepatic and cystic ducts in 12 patients (50%), no perforation site was observed in 9 patients (37.5%), and a common hepatic duct was observed in 3 patients (12.5%). All 24 patients underwent stage I surgery, and temporary biliary drainage was performed because of severe abdominal inflammation. Cholangiography and enhanced CT revealed an abnormal location of the pancreatic duct joining the bile duct in 64.3% patients. Following surgery, 15 patients underwent hepaticojejunostomy. Subsequently, 3-month to 6-year follow-up (median, 30 months) indicated that the patients recovered well with no serious complications.

**Conclusion:**

SBDP in children may be associated with pancreaticobiliary malunion (PBM) and congenital weakness of the bile duct wall. However, the clinical manifestations of this condition lack specificity; this limitation can be assisted through diagnosis *via* abdominal CT and by performing abdominal paracentesis. Once SBDP diagnosis is confirmed, the patient should follow the principles of individualized treatment.

Spontaneous bile duct perforation (SBDP) is a rare disease, mostly affecting children about 6 months of age, with the age at onset ranging from 25 weeks of gestation to 7 years after birth (1–3). The most common presentation of SBDP is abdominal distension, ascites, and jaundice; other symptoms may include localized or generalized peritonitis, pyrexia, and septic shock with or without signs and symptoms of a biliary tract disease (2, 4, 5).

The exact etiopathogenesis of SBDP is yet to be elucidated, although various theories have been proposed such as congenital weakness of the bile duct (1), distal biliary obstruction (6), and pancreaticobiliary duct anomalies (pancreaticobiliary malunion (PBM), etc.) (7).

Management strategies for SBDP are variable, ranging from non-operative management techniques [such as the use of broad-spectrum antibiotics, endoscopic retrograde cholangiopancreatography (ERCP), and percutaneous drainage] to complex surgical procedures (such as Roux-en-Y anastomosis) (8–14).

Given the infrequent nature of SBDP, the diverse management strategies, and the lack of defined outcomes, here, we conducted a study in 28 SBDP patients admitted to Wuhan Children's Hospital from January 2014 to January 2020 and reported the findings as follows.

## Materials and Methods

### General Information

In all, 28 patients with SBDP were admitted to Wuhan Children's Hospital from January 2014 to January 2020, 4 of whom were clinically diagnosed and 24 were confirmed by surgical exploration (male, 8; female, 20; male-to-female ratio, 1:2.5). The age at onset ranged from 6.5 months to 7.5 years, with the average age of 2.15 years. Informed consent forms were signed by the parents/guardians of all patients, and the study was approved by the Hospital Ethics Committee.

### Methods

In reference to the symptoms and signs, laboratory examinations, gallbladder ultrasound, and abdominal CT examinations were performed in the patients suspected with SBDP. In cases with difficulty in clinical diagnosis, patients underwent further abdominal paracentesis examination, and an SBDP diagnosis was clinically confirmed if abdominal paracentesis yielded biliary ascites. According to the caliber size of bile duct perforation during surgery, an appropriate surgical drainage scheme was determined. For patients with large perforated calibers (sufficiently large to place T-tubes), an extra-tube drainage of the bile ducts was administered. In patients with small (difficult to place T-tubes) or those without perforated calibers, a cholecystostomy tube was inserted instead. The subhepatic and pouch of Douglas drainage were added as necessary, and they were removed within 1 week if there was no drainage liquid and the abdominal ultrasound showed no ascites. When the T-tube drainage volume decreased, cholangiography was performed 2 weeks after surgery to observe the biliary tract, and the tube was clamped for 48 h. Both the biliary stent and external drain were removed only when the distal common bile duct showed no obstruction symptoms (e.g., abdominal pain, fever, and bile overflow). The patients were followed up regularly after surgery. If symptoms such as abdominal pain, fever, biliary dilatation, obstruction, and PBM (7, 15, 16) were confirmed, hepaticojejunostomy was performed.

## Results

### Clinical Manifestations

Most SBDP cases analyzed in this study were acute or subacute, and the time from onset to treatment was between 4 h and 15 days. The common symptoms were fever (24 cases, 85.7%), nausea and vomiting (22 cases, 78.6%), abdominal distension (19 cases, 67.9%), abdominal pain (15 cases, 53.6%), and clay-like stool (3 cases, 10.7%) (Table 1).

**Table 1:** Preoperative features of patients.

**Case**	**Complaint**			**Laboratory examination**			**CT**		**Diagnostic puncture**
	**Abdominal pain/distension**	**Emesis/Fever**	**Acholic stools**	**WBC** **(10**~**9/L)**	**NEU%**	**CRP** **(mg/l)**	**Bile duct dilatation**	**Ascites**	
1	+	+	–	N	61.5↑	10.3↑	+	+	+
2	–/+	–/+	–	N	53↑	12.2↑	+	+	Not done
3	+	+	–	33.05↑	84.2↑	24.2↑	+	+	Not done
4	+	+/–	–	N	77.7↑	45.1↑	+	+	+
5	+/–	–	–	17.37↑	85.3↑	83.9↑	–	+	+
6	–/+	+	+	N	N	3.86↑	–	+	+
7	–/+	+	–	17.59↑	62.9↑	13.8↑	+	+	Not done
8	+/–	+/–	–	14.95↑	56.4↑	20↑	+	+	+
9	+	+	–	19.1↑	85.4↑	63.2↑	+	+	+
10	–/+	–/+	–	13.23↑	59.9↑	46↑	+	+	+
11	+	+	–	14.2↑	77.8↑	126↑	+	+	+
12	+/–	+	–	12.25↑	68.6↑	45.9↑	+	+	–
13	+	+	–	16.91↑	82.4↑	57.9↑	+	+	+
14	–/+	+	–	24.4↑	75.2↑	80.5↑	–	+	+
15	+/–	+/–	–	11.35↑	82.6↑	N	+	+	+
16	–	+	–	12.4↑	68.6↑	133↑	–	+	+
17	–/+	+	–	16.25↑	87↑	6.19↑	+	+	+
18	–/+	–	+	14.41↑	N	44.7↑	+	+	+
19	+	+	–	N	68.7↑	45↑	+	+	+
20	–/+	+	+	13.49↑	N	4.32↑	+	+	+
21	+	+	–	14.5↑	87.5↑	75.4↑	–	+	+
22	–/+	+	–	11.19↑	N	69.3↑	+	+	+
23	–/+	+	+	N	81.6↑	143↑	–	+	–
24	+	+	–	20.68↑	81.7↑	89.8↑	–	+	+
25	–	+	–	26.99↑	75.3↑	155↑	+	+	Not done
26	–	–/+	–	18.61↑	74.8↑	50↑	+	+	Not done
27	+/–	–	–	12.2↑	86.5↑	N	+	+	Not done
28	+/–	+	–	18.36↑	74↑	44.7↑	+	+	+

*N: Indicates that the test value is within the normal range*.

*Case 1–24 underwent surgical exploration*.

*Case 25–28 recovered after conservative treatment*.

### Laboratory Examination

Of the 28 patients, 26 (92.9%) had elevated high-sensitivity C-reactive protein ranging from 3.86 to 155 mg/L, 24 (85.7%) had increased neutrophil count ranging from 53 to 87.5, 22 (78.6%) had exceptional peripheral blood leukocyte count ranging from 11.2 × 10^9^/L to 33.1 × 10^9^/L, 19 (67.9%) had increased serum total bilirubin level ranging from 24.4 to 731 μmol/L, 5 (17.9%) had raised conjugated bilirubin level ranging from 8.4 to 72.3 μmol/L, 8 (28.6%) had both raised alanine aminotransferase and aspartate aminotransferase levels, and 15 (53.6%) had high blood amylase levels (Table 1).

### Imaging Examination

**Figure 1:**
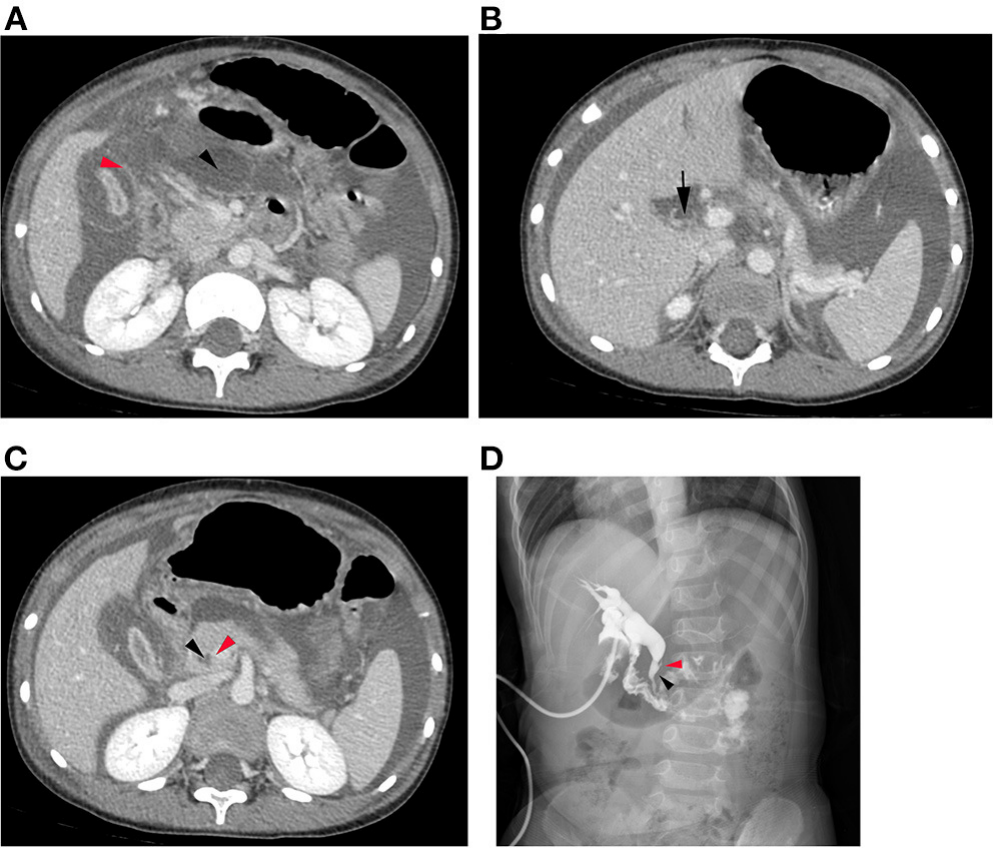
**(A)** Abdominal CT examination before surgery revealed thickened gallbladder wall with edema, narrow gallbladder cavity, and reinforced gallbladder mucosa (red arrow). Hydrops were found in the abdominal cavity and lesser omental bursa (black arrow). **(B)** A common bile duct rupture was observed in large perforations (black arrow). **(C)** Abdominal CT examination before surgery revealed that the pancreatic duct (red arrow) merged with the common bile duct (black arrow) in advance in cases with abnormal bile pancreatic confluence. **(D)** Cholangiography revealed that the pancreatic duct merged with the common bile duct in advance in cases with PBM.

The common imaging features of SBDP patients, such as thickened gallbladder wall with edema, void gallbladder cavity, and reinforced gallbladder mucosa (red arrow), were observed on abdominal CT examination before surgery. Ascites were found in the abdominal cavity and lesser omental bursa (black arrow) (Figure 1A). Furthermore, common bile duct rupture was observed in large perforations (Figure 1B). After surgical exploration, cholangiography revealed that the pancreatic duct merged with the common bile duct in advance in 18 patients (64.3%) with PBM (Figures 1C,D).

### Results of Abdominal Paracentesis and Surgical Exploration

Overall, 22 patients underwent abdominal paracentesis, and biliary ascites were discharged from the puncture point in 20 patients (Table 1). Furthermore, 24 patients underwent surgical exploration, and all of them were confirmed to have SBDP; their abdominal bile volumes ranged from 50 to 600 ml. Among these 24 cases, 12 had perforation sites at the confluence of the hepatic and cystic ducts, 9 had no visible perforation site, and 3 had a perforation site in the common hepatic duct. In the nine cases with no visible perforation site, eight underwent routine laparotomy or laparoscopic cholecystostomy. One patient (Case 14) underwent abdominal drainage. Furthermore, one patient (Case 1) was re-operated because of the presence of postoperative ascites; in this case, drainage tubes were placed in the omental foramen, subhepatic area, and splenic recess. One patient underwent laparoscopic common bile duct external T-tube drainage. Three patients had common hepatic duct perforations, one of whom opted for laparoscopic external T-tube drainage of the common bile duct, whereas the other two chose laparoscopy or laparoscopic cholecystostomy. Of these two patients, one (Case 21) underwent laparoscopic external choledochal T-tube drainage for the second time because of insufficient bile drainage on the third day after surgery. Among the 12 patients with perforation sites at the confluence of the common hepatic and cystic ducts, 8 opted for laparoscopy or laparoscopic cholecystostomy, whereas the other 4 underwent laparotomy or laparoscopic common bile duct external drainage (Table 2). Overall, 24 patients underwent cholangiography through T-tube or cholecystostomy tube for 1–3 months after surgery, and no obvious signs of obstruction were observed. After clamping the drainage tube for 48 h, drainage was removed only when the patient did not present with abdominal pain, fever, bile spillage, and other obstructive symptoms of the common bile duct.

**Table 2:** Operative data and follow-up situations of patients.

**Intraoperative situation**	**Diameter of bile common (cm)**	**Perforation site**	**Surgical procedure**			**Follow-up situation**		**Second operation**		**Time of the extraction of T-tube (month)**	**Follow-up time (month)**
			**Gallbladder drainage**	**Biliary drainage**	**(Intra-) peritoneal drainage**	**Diameter of bile duct**	**Observation time (month)**	**Laparoscopy**	**Laparotomy**		
1	3	Not found	+^a^		+^a^	2.5	7		+	/	6
2	0.8	Not found	+			2	1.5	+		/	19
3	0.8	CBD	+			2	4	+		/	23
4	1	CBD	+			3	3	+		/	23
5#	0.8	CHD	+			1.1	5	+		/	17
6	0.7	CBD		+		1.2	2	+		/	63
7	4	CBD	+			5	1.5	+		/	56
8	3	CBD		+		3	7	+		/	66
9	3	CBD	+			6	0.5	+		/	61
10	3	CBD	+			5	1	+		/	62
11	2.5	Not found		+		2	2	+		/	27
12	3	CBD	+			2.5	9	+		/	54
13	2.7	Not found	+			3.8	6	+		/	28
14	1.3	CBD	+		+	2	7	+		/	32
15	2	Not found	+			2.3	5	+		/	50
16	Unr	Not found	+			0.6		/	/	2	25
17	3	Not found	+			1.3		/	/	3	70
18	1.5	CHD		+		0.7		/	/	1	28
19	1.2	CBD		+		0.6		/	/	3	36
20	2.4	CBD	+			0.5		/	/	1	26
21	Unr	CHD	+^b^	+^b^		0.5		/	/	2	74
22	2.2	Not found	+			0.6		/	/	1.5	3
23	Unr	Not found	+			2.2		/	/	1.5	3
24	Unr	CBD		+		0.5		/	/	3	69

*CHD, common hepatic duct; CBD, common bile duct; Unr, Unrecorded*.

a *On this patient, the first operation was performed by laparoscopic gallbladder drainage, and on the 11th day after the operation, laparoscopic exploration was performed again because of the inadequate drainage, intraperitoneal drainage was respectively placed at the epiploic foramen, subhepatic region and splenic recess during the operation*.

b *On this patient, the first operation was performed by laparoscopic gallbladder drainage, and on the 3th day after the operation, laparoscopic bile duct drainage was performed again because of the inadequate drainage*.

# *This patient was hospitalized for cholangitis 1 month after surgery*.

### Follow-Up and Outcome

In 28 patients with SBDP, 4 were cured by conservative treatment and reported no discomfort and no obvious bile duct stenosis or dilatation *via* MRCP during follow-up period. After removal of the biliary drainage tube in 15 patients, the diameter of their common bile duct was 1.1–6.0 cm (confirmed by MRCP) after 1.5–9 months of observation. Among these patients, 14 underwent laparoscopic common bile duct cystectomy and common hepatic duct jejunum Roux-en-Y anastomosis, and 1 underwent open choledochal duct cystectomy and common hepatic duct jejunum Roux-en-Y anastomosis (Table 2). These 15 patients who underwent hepaticojejunostomy had presented with PBM, and common bile duct dilatation confirmed *via* imaging examination. After 6–66 months of follow-up, no patient reported any discomfort. Nine patients underwent simple external biliary drainage; the indwelling time of the drainage tube was 1–3 months (mean = 2 months). After 3–74 months of follow-up, the diameter of the patients' common bile duct was 0.5–2.2 cm, and they did not report any discomfort. PBM was observed in three out of these nine cases. Among these three cases, one had no common bile duct dilatation while the others had. Case 23 was lost to contact with a short-term follow-up time of only 3 months (Table 2).

## Discussion

Freedland reported the first case of SBDP in 1882 through an autopsy (17). SBDP usually affects children below the age of 4 years and is particularly reported in 6-month-old children. Studies have shown that 85% patients with SBDP are aged ≤2 years (3). In this study, more female than male patients were diagnosed with SBDP, with a ratio of 2.5:1. The minimum age at onset was 6.5 months, and the oldest patient was 7.5 years old. The average age was 2.15 years.

**Figure 2:**
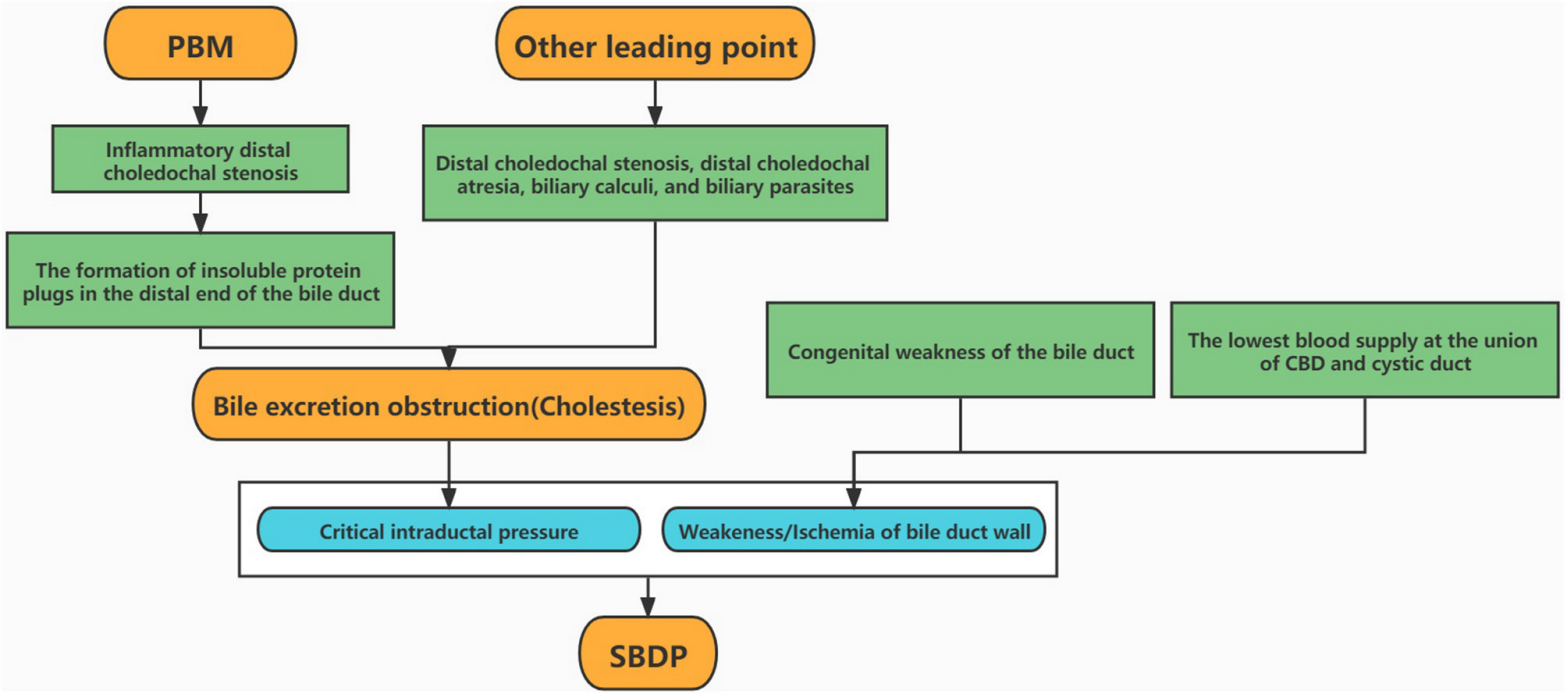
Theories of the mechanisms of SBDP.

At present, the exact pathogenesis of SBDP remains unclear, and it may be associated with various factors. SBDP is extremely rare in adults. Furthermore, acute pancreatitis, acalculous cholecystitis, human immunodeficiency virus infection, Hodgkin's lymphoma, tuberculosis, and severe necrotizing enterocolitis are considered important causative factors of SBDP in adults (18, 19). In contrast, health professionals believe that in the pediatric population, common bile duct dilation and SBDP may share a common etiology, i.e., PBM (7, 15, 16). In such cases, the convergence of pancreatic and bile ducts is abnormally located outside the duodenal wall (7). The abnormal confluence of pancreatic juice and bile leads to the formation of insoluble protein plugs in the distal end of the bile duct, in turn blocking the bile and pancreatic ducts and triggering abdominal pain (20, 21). In this study, 78.6% patients showed gastrointestinal symptoms, such as nausea and vomiting, and 53.6% showed clinical manifestations such as abdominal pain. Moreover, 53.6% patients underwent hepaticojejunostomy because of PBM and bile duct dilatation. Therefore, this study supports the clinical hypothesis that PBM is associated with SBDP. Distal choledochal stenosis, distal choledochal atresia, biliary calculi, and biliary parasites may cause cholestasis, which will then lead to inflammation, and increase the pressure on the bile duct, thereby increasing the possibility of SBDP. In this study, 24 patients with a higher number of biliary tract protein plugs during operation can support this view. Congenital weakness of the bile duct wall is another important factor observed among children with SBDP (1). Approximately, 60% of biliary blood supply comes from the posterior superior pancreaticoduodenal artery, whereas 38% originates from the hepatic artery. These blood vessels run along the course of the common bile duct. The union of the common bile duct and the cystic duct is seen at the end of the blood supply, which is the lowest point of blood supply. The union of the cystic duct and the common bile duct is therefore a congenital weak area of the biliary tract, and this is the main reason why SBDP usually develops here (22). In this study, intraoperative observation confirmed that the perforation site was at the union of the common hepatic and cystic ducts in half of the cases. This observation provided strong evidence for the theory of congenital weakness of the bile duct. In cases of abnormal union of pancreatic and bile ducts, pancreatic juice enters the biliary tract and causes local tissue damage. The pressure of the biliary tract suddenly rises because of biliary obstruction (cause by protein plugs), aggravating ischemia in the case of poor blood flow. These abovementioned factors work together to eventually cause SBDP (23) (Figure 2).

Pediatric SBDP lacks specific clinical manifestations. In this study, no evident peritoneal irritation sign was found in patients. The main reason is probably because bile leakage arising from biliary tract perforation was sterile. At an early stage, perforation causes sterile biliary peritonitis with inconspicuous peritoneal irritation. As the disease progresses and is combined with bacterial infection, non-specific symptoms such as fever, nausea, and vomiting are observed as the most common manifestations (24).

Laboratory and imaging examinations are required to assist SBDP diagnosis. Among the 28 patients in this study, 26 (92.9%) had elevated hypersensitive C-reactive protein, which was considerably higher than the proportion of patients with a high peripheral leukocyte count (78.6%). Thus, an increase in hypersensitive C-reactive protein can be used as an indicator for the early recognition of inflammation. In this study, 67.9% patients showed increased serum total bilirubin levels, but only 17.9% showed elevated conjugated bilirubin level; 28.6% patients had increased alanine aminotransferase and aspartate aminotransferase levels at the same time. Given that the perforation of the biliary tract can partially relieve cholestasis, no typical obstructive jaundice was observed. Therefore, the cases of SBDP should not be clinically measured using liver function indicators, such as increase in total bilirubin and conjugated bilirubin levels. In addition, 53.6% patients had elevated blood amylase levels; cholangiography and enhanced CT revealed the abnormal location of the joining of the pancreatic duct and the bile duct in 64.3% patients (Figures 1C,D). This result provided a basis for the clinical hypothesis that PBM is the leading point of SBDP.

Ultrasound is an important diagnostic tool in SBDP diagnostics. If abdominal ultrasound shows dilatation of the extrahepatic bile duct with ascites, then SBDP and bile peritonitis should be considered. The formation of limited fluid aggregation or pseudocysts (25) in and around the porta hepatis is also an important sign. However, SBDP is not easy to diagnose with only ascites and no bile duct expansion (21). In addition, ultrasound examination is limited by intestinal gases and restricted in the case of a non-expanding biliary system.

**Figure 3:**
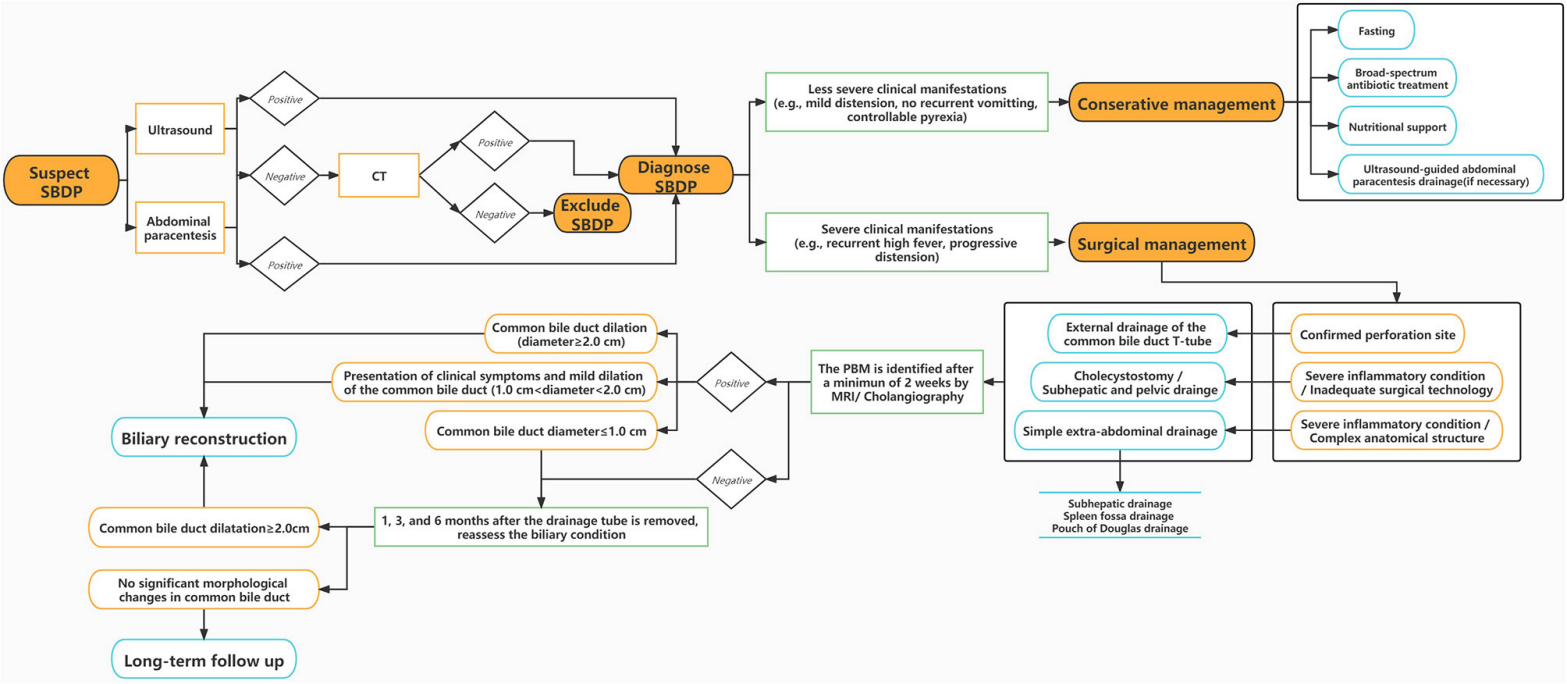
A single-center experience of managing SBDP.

Enhanced CT examination is as well a key diagnostic modality for SBDP diagnosis. SBDP often tends to manifest as cholecystitis and/or cholangitis, and their characteristics can be clearly distinguished on enhanced CT. The following two signs are highly suggestive of SBDP: (1) remarkably reduced gallbladder tension and wrinkled gallbladder wall accompanied by ascites, mainly around the gallbladder and (2) highly edematous gallbladder wall (1–2 cm water-like density band around the gallbladder), shrunken gallbladder cavity, and enhanced wrinkles in the inner mucous membrane (26). In addition, the breach can directly be observed when the perforation is large enough, providing strong evidence for SBDP diagnosis (Figure 3).

In resource-limited conditions, abdominal paracentesis is a practical alternative. In this study, 92.9% of patients successfully yielded yellow-brown ascites *via* abdominal paracentesis. These ascites had a higher bilirubin level (>6 mg/dl) than the normal range (0.7–0.8 mg/dl), suggesting the presence of bile ascites (27).

The therapeutic strategy for SBDP must be tailored to the situation. We reviewed 65 case reports or series published on SBDP, and the treatments were variable (Table 3 and Supplementary Table 1). In our study, four patients barely had symptoms of abdominal distension and vomiting and recovered successfully through fasting, antibiotic treatment, and nutritional support. In cases with large ascites, external drainage can be performed *via* ultrasound-guided abdominal paracentesis. Furthermore, abdominal distension is often a sign of paralytic intestinal obstruction, and a progressive increase in abdominal distension requires prompt surgery. The purpose of surgical treatment is to prevent persistent contamination by controlling bile leakage, draining the peritoneal cavity, and restoring biliary tract patency. The pathological causes of the biliary tract leading to perforation must be resolved as early as possible through laparotomy, laparoscopy, or robotic technology (28). Among the 24 patients who underwent surgery in our study, the choice between intraoperative cholecystostomy and common bile duct external T-tube drainage mainly depended on the intraoperative status of porta hepatis. When the perforation of the bile duct was large, common bile duct external T-tube drainage was performed. Bile leaks often cause severe inflammation in porta hepatis. Cholecystostomy is usually preferred because of its simplicity and for preventing damage to the surrounding blood vessels and porta hepatis. This procedure can be given priority at non-specialized hospitals or units with weaker hepatobiliary surgery technology. Successful SBDP treatment with the endoscopic placement of biliary stents has been recently reported in adult cases (1, 19, 29, 30), but not in children. In all cases, the abdominal cavity, especially subhepatic space (31), must be adequately flushed during surgery. However, after cholecystostomy, various problems may occur, such as insufficient bile drainage and ascites. To resolve this issue, multiple drainage tubes can be placed under the liver and pelvis to reduce the probability of a re-operation (7). Intraoperative repair of the perforation is unnecessary, and direct repair during surgery can easily lead to secondary injuries and postoperative stenosis (29). Under extreme conditions where the anatomical structure is unrecognizable because of severe abdominal inflammation, a simple extra-abdominal drainage can also be an option. In general, in this procedure, three drainage tubes are required to be placed under the liver, spleen fossa, and the pouch of Douglas for 3–4 weeks (31, 32).

**Table 3:** Location of biliary perforation in reported 132 cases.

**Report of SBDP**	
**Location of perforation**	***N*** **(%)**
Junction cystic duct-common hepatic duct	30 (22.7)
Cystic duct	22 (16.7)
Common bile duct	55 (41.7)
Hepatic duct	6 (4.5)
Gallbladder	9 (6.8)
No perforation site	10 (7.6)
Treatment	*N* (%)
Non-operative management	13 (9.8)
Surgical drainage	84 (63.6)
Biliary reconstruction	35 (26.5)

*The detailed of current studies is presented in Supplementary Table*.

Some researchers have reported distal obstruction caused by stones or stenosis in the distal bile duct to be a cause of perforation. However, others believe that obstruction may be a consequence of perforation, which results in slow bile transport and cholestasis, rather than being the cause of perforation. Obstruction is usually resolved by proper external biliary drainage (29, 33). Therefore, in the absence of intraoperative cholangiography, the best treatment is still cholecystostomy or T-tube drainage even if there is an obstruction in the distal biliary tract (34, 35).

Furthermore, patients with SBDP should be followed up regularly after surgery. In cases of recurrent abdominal pain, biliary dilatation, obstruction, and abnormal pancreaticobiliary duct confluence, biliary tract hepaticojejunostomy should be performed (17). Upadhyaya et al. (36) believed that if cholangiography (*via* T-tube or magnetic resonance cholangiopancreatography) suggests dilation of the common bile duct, hepaticojejunostomy should be performed to avoid biliary cirrhosis, portal hypertension, recurrent pancreatitis, and cholangiocarcinoma. However, hepaticojejunostomy must be performed after inflammation control. In this study, 24 patients underwent stage I surgery, and temporary external biliary drainage was performed because of severe abdominal inflammation. After operation, cases 5 and 6 exhibited persistent abdominal pain and discomfort. Abdominal CT revealed that their biliary tract was dilated to varying degrees (1.1 and 1.2 cm, respectively). Furthermore, hepaticojejunostomy in cases 1–4 and 7–15 was performed when the dilation of the common bile duct was over 2.0 cm, and good recovery was observed in these cases. The dilation of the common bile duct in cases 16, 18–22, and 24 was within 1.0 cm. Surprisingly, although biliary dilatation was observed in cases 17 and 23 (especially case 23, where the diameter of the common bile duct reached 2.2 cm) after the surgery, they did not have any postoperative complications, which indicated that hepaticojejunostomy was unnecessary.

Existing studies have indicated that SBDP and congenital biliary dilatation (CBD) share the same etiology (8, 37). Thus, we suggest that PBM is the key in the determination of whether to perform hepaticojejunostomy. In our experience, in SBDP patients with biliary dilatation after surgery, hepaticojejunostomy may temporarily not be performed if the patient does not experience persistent or recurrent abdominal pain, fever, and other symptoms. The patients can simply be instructed to visit for regular follow-ups, and hepaticojejunostomy can be actively performed if the aforementioned symptoms occur. Besides, if hepaticojejunostomy is performed in asymptomatic patients with a common bile duct diameter of ≤1.0 cm, the difficulty of biliary and intestinal anastomosis will be increased. We reckon that hepaticojejunostomy should be performed if the following conditions exist (Figure 3): (1) common bile duct dilation (diameter ≥2.0 cm); (2) presentation of clinical symptoms and mild dilation of the common bile duct (1.0 cm < diameter <2.0 cm) combined with PBM; and (3) no peritonitis 2–8 weeks after SBDP external drainage.

There are some limitations to the current study. The current study reports the experience of a single pediatric tertiary center. Although our study is the largest study of SDBP to date, a total of 28 cases are still insufficient. Further analysis of follow-up information was limited by insufficient follow-up time, especially for patients with a common bile duct diameter of ≤1.0 and >2.0 cm but no hepaticojejunostomy. Furthermore, it is currently unclear whether the bile ducts will continue to expand or increase the risk of carcinogenesis. Further studies need to be conducted to evaluate these factors in detail.

Finally, based on our experience, three treatments should be followed for SBDP treatment (Figure 3): (1) nonsurgical treatment, such as antibiotic treatment, and B-ultrasound-guided external drainage by abdominal paracentesis; (2) laparotomy or laparoscopy and drainage through cholecystostomy tube or biliary T-tube; and (3) biliary tract reconstruction by laparoscopic or open hepaticojejunostomy in phase II. Conservative treatment is given priority for SBDP in clinical practice. Furthermore, external biliary drainage (temporary cholecystostomy or external biliary drainage through T-tube) with or without subhepatic drainage can be performed to promote spontaneous closure of the biliary tract perforation. Finally, hepaticojejunostomy should only be considered for patients with malformations of pancreaticobiliary duct or common bile duct dilatation (30).

## Data Availability Statement

The original contributions presented in the study are included in the article/Supplementary Material, further inquiries can be directed to the corresponding author.

## Ethics Statement

The studies involving human participants were reviewed and approved by Wuhan Children's Hospital Ethics Committee. Written informed consent to participate in this study was provided by the participants' legal guardian/next of kin.

## Author Contributions

All authors listed have made a substantial, direct, and intellectual contribution to the work and approved it for publication.

## Conflict of Interest

The authors declare that the research was conducted in the absence of any commercial or financial relationships that could be construed as a potential conflict of interest.

## Publisher's Note

All claims expressed in this article are solely those of the authors and do not necessarily represent those of their affiliated organizations, or those of the publisher, the editors and the reviewers. Any product that may be evaluated in this article, or claim that may be made by its manufacturer, is not guaranteed or endorsed by the publisher.
