# Supplementary material for: Analysis of the Clinical Characteristics of Spontaneous Bile Duct Perforation in Children
Source: Front Pediatr. 2022 Mar 23;10:799524. doi: 10.3389/fped.2022.799524 (PMC8984294; doi:10.3389/fped.2022.799524)
Supplement: Supplementary file 1 [file Data_Sheet_1.DOCX]

Supplementary table: published study in SBDP

| Year | Country | Investigator  (s) | Case  (s) | Perforation site | Probable aetiology | treatments |
| --- | --- | --- | --- | --- | --- | --- |
| 2020 | Mexico | Godínez-Borrego (et al.) | 1 | common bile duct | - | Roux-en-Y hepaticojejunostomy |
| 2018 | India | Sunil K(et al.) | 1 | common hepatic duct | - | T-tube insertion |
| 2016 | Lahore-Pakistan | Malik, H.S (et al.) | 22 | common bile duct | - | percutaneous drainage of cyst :5  t-tube insertion :10  Cholecystectomy :7 |
| 2016 | India | C. Sharma(et al.) | 1 | common bile duct | - | French stent |
| 2015 | India | Kurbet, S.B (et al.) | 1 | common hepatic duct | weakness of the biliary canal wall  during embryogenesis with pancreaticobiliary malunion | Roux-en-Y hepatojejunostomy |
| 2014 | USA | Jeanty C(et al.) | 4 | the junction of the cystic and common hepatic ducts: 3  Gallbladder: 1 | - | external drain at the porta hepatis |
| 2013 | India | Upadhyaya, V.D(et al.) | 6 | common bile duct: 3  cystic duct: 1  no perforation site: 2 | - | T-tube insertion:3  Exteral drain:3 |
| 2013 | USA | J.T. Murphy(et al.) | 3 | cystic duct: 2  no perforation site：1 | - | cholecystostomy tube and peri  portal drains |
| 2012 | Australia | Pereira (et al.) | 1 | common bile duct | - | Cholecystostomy |
| 2012 | China,taiwan | Chen(et al.) | 1 | the junction of the cystic and common hepatic ducts | - | Roux-en-Y hepaticojejunostomy |
| 2012 | Iran | Joodi(et al.) | 1 | cystic duct | - | drainage tubes 、Delayed repairing of the perforation |
| 2012 | India | Satish(et al.) | 1 | cystic duct | - | external drain |
| 2011 | India | Shukla(et al.) | 4 | gallbladder | inflammatory reaction | cholecystectomy |
| 2011 | Italy | Gobbi(et al.) | 1 | cystic duct | - | conservative approach |
| 2010 | Korea | Lee(et al.) | 3 | the junction of the cystic and common hepatic ducts | - | drainage |
| 2010 | UK | Evans K. (et al.) | 2 | cystic duct | Congenital weakness of the bile duct wall | cholecystectomy +T tube：1  an external drain：1 |
| 2009 | India | V. Jadhav , R(et al.) | 1 | cystic duct | - | biliary stenting |
| 2008 | China，Taiwan | Lu Y Y(et al.) | 1 | gallbladder | splanchnic ischemia | Cholecystectomy |
| 2008 | UK | Davenport(et al.) | 2 | the junction of the cystic and common hepatic ducts | - | reconstruction hepaticojejun  ostomy. |
| 2008 | India | Vijay(et al.) | 1 | cystic duct | - | Cholecystectomy |
| 2007 | Bangalore | K. Das(et al.) | 1 | common bile duct | - | T-tube drainage |
| 2007 | India | Kanojia(et al.) | 3 | cystic duct | - | T-tube drainage |
| 2007 | Tunisia | Sahnoun(et al.) | 1 | common bile duct | - | cholecystostomy |
| 2006 | Singapore | S P.TR(et al.) | 2 | common bile ductct：1  no perforation site：1 | - | Roux-en-Y hepaticojejunostomy |
| 2006 | Israel | ZMD(et al.) | 1 | the junction of the cystic and common hepatic ducts | abnormal bile pancreatic junction | T-tube insertion |
| 2006 | USA | Barnes B H(et al.) | 1 | the junction of the cystic and common hepatic ducts | Reflux of pancreatic secretions  weakness of the bile duct wall | 5-cm 7F internal biliary stent |
| 2006 | Iran | Imanieh(et al.) | 1 | cystic duct | - | T-tube insertion |
| 2006 | UK | Davenport(et al.) | 1 | common bile duct | unknown | Roux-en-y hepaticojejunostomy |
| 2005 | UK | Gull s(et al.) | 1 | gallbladder neck | a combination of maternal infection and perinatal asphyxia | direct repair |
| 2004 | - | Sharma(et al.) | 1 | gallbladder | - | cholecystectomy |
| 2003 | USA | Xanthakos, S.A (et al.) | 1 | common bile duct | - | an intraabdominal drain |
| 2003 | India | Sharma(et al.) | 2 | at the junction of the cystic duct and common hepatic ducts：1  no perforation site：1 | - | Conservative treatment：1  Cholecystostomy biliary drainage：1 |
| 2002 | Portugal | Costa(et al.) | 1 | gallbladder | secondary to obstruction | Cholecystectomy |
| 2002 | Hongkong | W.T. Ng(et al.) | 1 | common bile duct | - | ERCP with stent placement |
| 2001 | India | Kumar(et al.) | 1 | no perforation site | - | Roux- en- Y  cholecystojejunostomy |
| 2001 | India | Kasat, L.S (et al.) | 1 | common bile duct | - | Peritoneal drainage tube |
| 2000 | USA | Goldberg, D (et al.) | 1 | the junction of the cystic and common hepatic ducts | Congenital weakness of the bile duct wall | Cholecystostomy+external drainage of the biliaryperforation |
| 2000 | Japan | Hasegawa, T (et al.) | 3 | at the junction of the cystic and common hepatic ducts. | - | T-tube insertion |
| 2000 | Turkey | Meltem(et al.) | 1 | common hepatic duct | - | drain |
| 2000 | India | Prabakaran(et al.) | 1 | at the junction of the cystic and common hepatic ducts | - | suture |
| 1999 | USA | CECCECILIA MIA M(et al.) | 1 | common bile duct | - | cholecystostomy |
| 1999 | Spain | Pradas(et al.) | 1 | common hepatic duct | - | Roux-en-Y hepatojejunostomy |
| 1998 | UK | Mirajkar(et al.) | 1 | cystic duct | - | cholecystectomy |
| 1996 | France | Clwrdot C(et al.) | 11 | Common hepatic duct:2  Cystic duct:2  Junction of the cystic duct and Hepatic ducts:4  common bile duct:2  no perforation site：1 | Panereatico-biliary reflux | Biliary reconstructlon:5  External drain :4  Cholecystectomy:1  Spontaneous recovery:1 |
| 1996 | USA | Spigland(et al.) | 2 | common bile duct | - | Cholecystostomy |
| 1996 | Saudi Arabia | Souheil(et al.) | 1 | at the junction of the cystic duct and common hepatic ducts | - | choledochoduodenostomy |
| 1995 | USA | Rosen(et al.) | 1 | common bile duct | - | Repair |
| 1995 | UK | Hirigoyen,M B  (et al.) | 1 | common bile duct | - | T tube |
| 1993 | UK | Smethurst, F.A (et al.) | 1 | common bile duct. | - | Reconstruction of the biliary tract |
| 1993 | Iran. | Banani, S.A (et al.) | 3 | common bile duct:2  cystic ducts:1 | - | A Penrose drain was put under the liver：1  cholecystostomy ：1  Suture：1 |
| 1991 | UK | Davenport  (et al.) | 6 | at or near the junction of the cystic with the common hepatic duct | Inspissated bile obstruction | Cholecystectomy and  latex T tube drainage ：2  Roux-en-y hepaticojejunostomy  ：2  Roux-en-y cholecystoenterostomy ：2 |
| 1991 | USA | Dolgin S E(et al.) | 1 | common bile duct | ischemia associated with neonatal necrotizing enterocolitis may have weakened the common bile duct near the duodenum | T-tube |
| 1990 | USA | Saltzman(et al.) | 1 | at the junction of the cystic duct and common hepatic ducts | congenital develop  mental biliary tract  abnormality | Roux-en-Y hepaticojejunostomy |
| 1990 | USA | Chilukuri s(et al.) | 1 | common bile duct | a localized embryonic mu  ral malformation | cholecystostomy |
| 1988 | Australia | A.Shun(et al.) | 1 | cystic duct | - | T-tube insertion |
| 1986 | USA | Bahia, J.O (et al.) | 1 | at the junction of the cystic and common hepatic ducts. | - | A cholecystostomy tube +infrahepatic drains |
| 1983 | Japan | Yano, H (et al.) | 3 | common bile duct:2  no perforation site:1 | abnormal bile pancreatic junction | Drainage tubes  Choledocho-duodenostomy |
| 1982 | Japan | Y.Yamashiro(et al.) | 2 | no perforation site | abnormal bile pancreatic junction | Choledochocysto-duodenostomy |
| 1980 | South Africa | Lloyd, D.A (et al.) | 4 | common bile duct :2  Cystic duct:2 | - | Choledochocysto-duodenostomy |
| 1978 | England | J.B.Witcombe(et al.) | 1 | common bile duct | Roundworm infection | Cholecystostomy |
| 1977 | Japan | H. Ohkawa(et al.) | 3 | common bile duct | abnormal bile pancreatic junction | A small T-tube was placed in the common bile duct through the perforation. |
| 1972 | - | N Nadaraja(et al.) | 1 | the cystic ducts. | - | a T-tube placed through the perforation. |

Reference：

[1]. Bahia, J.O., D.K. Boal, S.R. Karl, and G.W. Gross, *Ultrasonographic detection of spontaneous perforation of the extrahepatic bile ducts in infancy.* Pediatr Radiol, 1986. **16**(2): p. 157-9.

[2]. Banani, S.A., A. Bahador, and N. Nezakatgoo, *Idiopathic perforation of the extrahepatic bile duct in infancy: pathogenesis, diagnosis, and management.* J Pediatr Surg, 1993. **28**(7): p. 950-2.

[3]. Chen, T.Z., H.C. Chen, and C.M. Chou, *Spontaneous perforation of the bile duct in a neonate: drainage or resection?* J Chin Med Assoc, 2012. **75**(7): p. 353-4.

[4]. Evans, K., N. Marsden, and A. Desai, *Spontaneous perforation of the bile duct in infancy and childhood: a systematic review.* J Pediatr Gastroenterol Nutr, 2010. **50**(6): p. 677-81.

[5]. Godínez-Borrego, C.G., S. Velasco-Villanueva, and J.A. Mújica-Guevara, *Spontaneous perforation of the common bile duct in a pediatric patient. Case report and short review of the literature.* Cir Cir, 2020. **88**(2): p. 211-214.

[6]. Goldberg, D., D. Rosenfeld, and S. Underberg-Davis, *Spontaneous biliary perforation: biloma resembling a small bowel duplication cyst.* J Pediatr Gastroenterol Nutr, 2000. **31**(2): p. 201-3.

[7]. Hasegawa, T., Y. Udatsu, M. Kamiyama, T. Kimura, et al., *Does pancreatico-biliary maljunction play a role in spontaneous perforation of the bile duct in children?* Pediatr Surg Int, 2000. **16**(8): p. 550-3.

[8]. Jeanty, C., S.C. Derderian, S. Hirose, H. Lee, et al., *Spontaneous biliary perforation in infancy: Management strategies and outcomes.* J Pediatr Surg, 2015. **50**(7): p. 1137-41.

[9]. Kasat, L.S., S.S. Borwankar, M. Jain, and A. Naregal, *Spontaneous perforation of the extrahepatic bile duct in an infant.* Pediatr Surg Int, 2001. **17**(5-6): p. 463-4.

[10]. Kumar, V., A. Chattopadhyay, N. Bhat, and P.L. Rao, *Spontaneous biliary perforation presenting as gastric outlet obstruction.* Indian J Pediatr, 2001. **68**(4): p. 361-3.

[11]. Kurbet, S.B., G.P. Prashanth, V.D. Patil, and R.M. Wali, *Intact choledochal cyst with spontaneous common hepatic duct perforation: a spectrum of congenital biliary canal defects?* J Pediatr Gastroenterol Nutr, 2015. **60**(1): p. e1.

[12]. Lee, M.J., M.J. Kim, and C.S. Yoon, *MR cholangiopancreatography findings in children with spontaneous bile duct perforation.* Pediatr Radiol, 2010. **40**(5): p. 687-92.

[13]. Livesey, E. and M. Davenport, *Spontaneous perforation of the biliary tract and portal vein thrombosis in infancy.* Pediatr Surg Int, 2008. **24**(3): p. 357-9.

[14]. Lloyd, D.A. and R.E. Mickel, *Spontaneous perforation of the extra-hepatic bile ducts in neonates and infants.* Br J Surg, 1980. **67**(9): p. 621-3.

[15]. Malik, H.S., H.A. Cheema, Z. Fayyaz, M.A. Hashmi, et al., *Spontaneous Perforation Of Bile Duct, Clinical Presentation, Laboratory Work Up, Treatment And Outcome.* J Ayub Med Coll Abbottabad, 2016. **28**(3): p. 518-522.

[16]. Murphy, J.T., K. Koral, T. Soeken, and S. Megison, *Complex spontaneous bile duct perforation: an alternative approach to standard porta hepatis drainage therapy.* J Pediatr Surg, 2013. **48**(4): p. 893-8.

[17]. Nadaraja, N., *Spontaneous perforation of the common bile duct in a neonate.* J Pediatr Surg, 1972. **7**(3): p. 340-1.

[18]. Nyamannawar, B.M. and K. Das, *Spontaneous infantile choledochal cyst perforation.* Indian J Pediatr, 2007. **74**(3): p. 299-300.

[19]. Ohkawa, H., H. Takahashi, and M. Maie, *A malformation of the pancreatico-biliary system as a cause of perforation of the biliary tract in childhood.* J Pediatr Surg, 1977. **12**(4): p. 541-6.

[20]. Pereira, E.C.M.V., J. Yan, M. Asaid, P. Ferguson, et al., *Conservative management of spontaneous bile duct perforation in infancy:: case report and literature review.* J Pediatr Surg, 2012. **47**(9): p. 1757-9.

[21]. Sai Prasad, T.R., C.H. Chui, Y. Low, C.L. Chong, et al., *Bile duct perforation in children: is it truly spontaneous?* Ann Acad Med Singap, 2006. **35**(12): p. 905-8.

[22]. Sharma, C., J. Desale, M. Waghmare, and H. Shah, *A Case of Biliary Peritonitis following Spontaneous Common Bile Duct Perforation in a Child.* Euroasian J Hepatogastroenterol, 2016. **6**(2): p. 167-169.

[23]. Shun, A., A.W. Middleton, K.R. Kamath, and D. Brewster, *Spontaneous perforation of common bile duct.* J Pediatr Gastroenterol Nutr, 1988. **7**(1): p. 132-4.

[24]. Smethurst, F.A. and H. Carty, *Case report: spontaneous perforation of the common bile duct in infancy.* Br J Radiol, 1993. **66**(786): p. 556-7.

[25]. Steiner, Z. and D. Dimitrov, *Spontaneous perforation of the common bile duct mimicking choledochal cyst.* Isr Med Assoc J, 2006. **8**(9): p. 655-6.

[26]. Sunil, K., A. Gupta, A.K. Verma, A.K. Singh, et al., *Spontaneous common hepatic duct perforation in a child: A rare case report.* Afr J Paediatr Surg, 2018. **15**(1): p. 53-55.

[27]. Upadhyaya, V.D., B. Kumar, M. Singh, Rudramani, et al., *Spontaneous biliary peritonitis: Is bed side diagnosis possible?* Afr J Paediatr Surg, 2013. **10**(2): p. 112-6.

[28]. Witcombe, J.B., *Ascaris perforation of the common bile duct demonstrated by intravenous cholangiography.* Pediatr Radiol, 1978. **7**(2): p. 124-5.

[29]. Xanthakos, S.A., N.A. Yazigi, F.C. Ryckman, and M.S. Arkovitz, *Spontaneous perforation of the bile duct in infancy: a rare but important cause of irritability and abdominal distension.* J Pediatr Gastroenterol Nutr, 2003. **36**(2): p. 287-91.

[30]. Yamashiro, Y., M. Sato, and A. Hoshino, *Spontaneous perforation of a choledochal cyst.* Eur J Pediatr, 1982. **138**(2): p. 193-5.

[31]. Yano, H. and H. Matsumoto, *Choledochal cyst following operation for idiopathic perforation of the biliary tract in childhood.* Jpn J Surg, 1983. **13**(5): p. 441-5.

[32]. Carubelli, C.M. and T.J. Abramo, *Abdominal distention and shock in an infant.* Am J Emerg Med, 1999. **17**(4): p. 342-4.

[33]. Chilukuri, S., V. Bonet, and M. Cobb, *Antenatal spontaneous perforation of the extrahepatic biliary tree.* American Journal of Obstetrics and Gynecology, 1990. **163**(4): p. 1201-1202.

[34]. Dolgin, S.E., R.L. Levine, K.I. Norton, J.R. Marolda, et al., *Complete spontaneous disruption of the common bile duct: a late complication of necrotizing enterocolitis?* J Pediatr Gastroenterol Nutr, 1991. **12**(3): p. 379-82.

[35]. Gobbi, D., F.F. Leon, P. Gasparella, P. Gamba, et al., *Conservative treatment of spontaneous biliary perforation.* Pediatrics International, 2011. **53**(4): p. 594-595.

[36]. Jadhav, V., R. Gupta, S.V. Parelkar, H. Shah, et al., *Endoscopic Management of Spontaneous Perforation of a Cystic Duct in a 10-year-old Child.* European Journal of Pediatric Surgery, 2009. **19**(03): p. 194-196.

[37]. Makin, E. and M. Davenport, *Idiopathic biliary perforation in a 10-year-old boy.* Pediatr Surg Int, 2006. **22**(5): p. 465-7.

[38]. Bingol-Kologlu, M., I. Karnak, T. Ocal, and F.C. Tanyel, *Idiopathic perforation of the bile duct in an infant.* J Pediatr Gastroenterol Nutr, 2000. **31**(1): p. 83-5.

[39]. Estevao-Costa, J., M. Soares-Oliveira, J.M. Lopes, and J.L. Carvalho, *Idiopathic perforation of the gallbladder: a novel differential diagnosis of acute abdomen.* J Pediatr Gastroenterol Nutr, 2002. **35**(1): p. 88-9.

[40]. W, N., C. C, and C. S, *Is spontaneous perforation of the bile duct in children due solely to pancreatico-biliary maljunction?* Pediatric Surgery International, 2002. **18**(5-6): p. 565-566.

[41]. Lu, Y.Y., H.S. Lai, W.S. Hsieh, and W.M. Hsu, *Ischemic gallbladder perforation in a premature infant.* J Pediatr Surg, 2008. **43**(6): p. E31-2.

[42]. Ibanez, D.V., J.J. Vila, M.S. Fernandez, I. Guemes, et al., *Spontaneous biliary perforation and necrotizing enterocolitis.* Pediatr Surg Int, 1999. **15**(5-6): p. 401-2.

[43]. Prabakaran, S., N. Kumaran, S.R. Regunanthan, N. Prasad, et al., *Spontaneous biliary perforation in a child with features of Ivemark syndrome.* Pediatr Surg Int, 2000. **16**(1-2): p. 109-10.

[44]. Kanojia, R.P., S.K. Sinha, J. Rawat, A. Wakhlu, et al., *Spontaneous biliary perforation in infancy and childhood: clues to diagnosis.* Indian J Pediatr, 2007. **74**(5): p. 509-10.

[45]. Gull, S., M. Singh, and J. Bruce, *Spontaneous gallbladder perforation in a neonate.* Pediatric Surgery International, 2005. **21**(8): p. 657-658.

[46]. Joodi, M., N. Norouzbeigi, M.A. Rad, R. Shojaeian, et al., *Spontaneous perforation of common bile duct in a pediatric patient: application of hepatobiliary scintigraphy.* Clin Nucl Med, 2012. **37**(10): p. 1006-8.

[47]. Barnes, B.H., M.R. Narkewicz, and R.J. Sokol, *Spontaneous perforation of the bile duct in a toddler: the role of endoscopic retrograde cholangiopancreatography in diagnosis and therapy.* J Pediatr Gastroenterol Nutr, 2006. **43**(5): p. 695-7.

[48]. Hirigoyen, M.B., J.G. Geoghegan, E.R. Owen, and M.P. Singh, *Spontaneous perforation of the bile duct in infancy.* Eur J Pediatr Surg, 1995. **5**(6): p. 375-6.

[59]. Davenport, M., N.D. Heaton, and E.R. Howard, *Spontaneous perforation of the bile duct in infants.* Br J Surg, 1991. **78**(9): p. 1068-70.

[50]. Chardot, C., F. Iskandarani, O. De Dreuzy, B. Duquesne, et al., *Spontaneous perforation of the biliary tract in infancy: a series of 11 cases.* Eur J Pediatr Surg, 1996. **6**(6): p. 341-6.

[51]. Imanieh, M.H., A. Mowla, D. Zohouri, H.R. Forootan, et al., *Spontaneous perforation of the common bile duct with eosinophilia in an 18-month-old girl: a case report and review of literature.* Med Sci Monit, 2006. **12**(4): p. CS31-3.

[52]. Rosen, J.M., *Hepatobiliary scintigraphy in spontaneous perforation of the common bile duct.* Clin Nucl Med, 1995. **20**(2): p. 187-8.

[53]. Satish, J., J. Monica, K. Dalbir, and S. Lovesh, *Management of spontaneous perforation of the bile duct in an infant in a semi-urban setup: a case report.* Malays J Med Sci, 2012. **19**(1): p. 73-5.

[54]. Shabib, S.M., A. al-Rabeeah, A. Rifai, and A. Telmesani, *Spontaneous bile duct perforation associated with fatty liver in an infant.* J Pediatr Gastroenterol Nutr, 1996. **23**(4): p. 466-9.

[55]. Vijay, B.B., R. Kumar, D.K. Gupta, M. Ragavan, et al., *Spontaneous biliary perforation in an infant: an unusual chronic presentation.* Clin Nucl Med, 2008. **33**(4): p. 273-5.

[56]. Sharma, S.B., S.C. Sharma, and V. Gupta, *Spontaneous biliary perforation: a rare entity in late infancy and childhood.* Indian J Pediatr, 2003. **70**(10): p. 829-31.

[57]. Spigland, N., R. Greco, and D. Rosenfeld, *Spontaneous biliary perforation: does external drainage constitute adequate therapy?* J Pediatr Surg, 1996. **31**(6): p. 782-4.

[58]. Shukla, R.M., D. Roy, P.P. Mukherjee, K. Saha, et al., *Spontaneous gall bladder perforation: a rare condition in the differential diagnosis of acute abdomen in children.* J Pediatr Surg, 2011. **46**(1): p. 241-3.

[59]. Suresh-Babu, M.V., A.G. Thomas, V. Miller, and A. Dickson, *Spontaneous perforation of the cystic duct.* J Pediatr Gastroenterol Nutr, 1998. **26**(4): p. 461-3.

[60]. Sahnoun, L., M. Belghith, M. Jallouli, K. Maazoun, et al., *Spontaneous perforation of the extrahepatic bile duct in infancy: report of two cases and literature review.* European Journal of Pediatrics, 2006. **166**(2): p. 173-175.

[61]. Saltzman, D.A., C.L. Snyder, and A. Leonard, *Spontaneous perforation of the extrahepatic biliary tree in infancy. A case report.* Clin Pediatr (Phila), 1990. **29**(6): p. 322-4.

[62] Sharma SB, Gupta V, Sharma V. Spontaneous gall bladder perforation: a rare entity in infants. Indian J

Gastroenterol 2004;23:75–6.
